# Enhancing the Buckling Performance of Thin-Walled Plastic Structures Through Material Optimization

**DOI:** 10.3390/polym17192697

**Published:** 2025-10-07

**Authors:** Alexander Busch, Olaf Bruch, Dirk Reith

**Affiliations:** 1 Institute of Technology, Resource and Energy-Efficient Engineering (TREE), Bonn-Rhein-Sieg University of Applied Sciences, Grantham-Allee 20, 53757 Sankt Augustin, Germany; olaf.bruch@h-brs.de; 2 Fraunhofer Institute for Algorithms and Scientific Computing (SCAI), Schloss Birlinghoven, 53754 Sankt Augustin, Germany; dirk.reith@h-brs.de

**Keywords:** sensitivity-based optimization, enhancing buckling resistance, thin-walled structures, extrusion blow molding, nonlinear structural optimization, high-density polyethylene

## Abstract

Reducing material usage in plastic products is a key lever for improving resource efficiency and minimizing environmental impact. In thin-walled structures subjected to mechanical loading, material efficiency must be achieved without compromising structural performance. In particular, resistance to buckling, a critical failure mode, must be taken into account during product development. Due to the large number of design and process variables, many of which are interdependent, optimization approaches are uncommon in the blow-molded packaging industry. This paper presents a sensitivity-based optimization approach to improve buckling resistance by modifying the product’s material distribution. Since the sensitivity is nonlinear and depends on the product’s deformation state, various methods are developed and tested to reduce the frame-wise sensitivity data to a single sensitivity vector suitable for optimization. These methods are then tested on common extrusion blow-molded products, achieving improvements in buckling load of up to 60%. This approach is transferable to other thin-walled structures across various engineering domains, offering a pathway toward lightweight yet load-compliant designs.

## 1. Introduction

In the development of loaded, thin-walled structures, such as extrusion blow-molded packaging products, the sudden onset of buckling, and therefore the sudden loss of stability, is the principal failure mode. Designing structures that can withstand the highest possible load before buckling remains a central challenge for product developers. Buckling resistance primarily depends on geometry and wall thickness. Since the geometry is often constrained by functional or aesthetic requirements, as well as the tool geometry, wall thickness typically serves as the main adjustable parameter for improving stability.

Experience shows that concentrating wall thickness at corners is generally an effective way to increase structural capacity. Another approach is to reinforce the areas most susceptible to buckling. While reinforcement at corners is straightforward, reinforcing buckling-sensitive regions is inherently more complex, as the critical areas are not easily identifiable and must be determined through simulation. Moreover, as the simulation progresses and the product deforms, these regions may shift, adding further complexity. Defining the optimal wall thickness is therefore a challenging task, particularly when restrictions on the maximum allowable product weight apply or symmetry is enforced. Taken together, these factors make the optimization of wall thickness distribution in thin-walled products under load a highly complex task.

Despite these challenges, optimization offers a promising pathway to reduce product weight without compromising performance. By increasing buckling resistance through optimized wall thickness distribution, products can be designed with less material, directly improving resource efficiency and reducing environmental impact, a goal increasingly prioritized across industries.

Because the sensitivity of the buckling load to the wall thickness depends strongly on the product’s deformation state, and is therefore a transient quantity, a critical question arises: at which deformation stage should sensitivities be evaluated to most effectively improve load-bearing capacity? This study addresses that question and introduces several strategies to exploit sensitivities across different deformation stages.

This paper presents methods to filter the transient sensitivity data, so that they are reduced to a vector of scalar values and are accessible for sensitivity-based optimization algorithms. Furthermore, an optimization workflow is developed in order to test the different schemes to filter the sensitivity data. The practical applicability of the filtering schemes is evaluated using the optimization workflow together with typical products of the extrusion blow molding process. Among the strategies evaluated, the most effective approaches improved the buckling load by up to 60%, clearly showing the potential of the method.

While the developed workflow is demonstrated with blow-molded packaging containers, buckling occurs in a wide range of engineering applications, including thin composite structures, wind turbine blades, and other lightweight components. The underlying principles of optimizing structural stability are therefore broadly applicable across disciplines.

In the following, the blow molding processes is briefly introduced. Subsequently, current methodologies for simulating and optimizing blow-molded packaging products are presented.

### 1.1. The Blow Molding Process

Extrusion blow molding [1] is a manufacturing process for producing hollow plastic products. A plastic tube, known as a parison, is extruded and subsequently inflated inside a mold until it conforms to the cavity shape, cools down, and is then demolded and post-processed to obtain the final product. The wall thickness of the finished part depends on both the parison’s initial thickness and the local stretch ratio required to fill the mold geometry. The parison’s wall thickness distribution can be actively controlled in the axial, circumferential, or radial direction, depending on the applied wall thickness control strategy.

A common method is Axial Wall Thickness Control, in which the extrusion die opening is dynamically adjusted to uniformly influence the thickness along the parison’s axis. Another technique, Partial Wall Thickness Control, involves dynamic deformation of the outer parison die during extrusion. This produces a non-uniform thickness distribution, enabling the strategic placement of thicker or thinner sections around the parison circumference [2].

In contrast to these dynamic approaches, static modifications alter the nozzle gap geometry before extrusion. Such modifications generate a radial wall thickness profile that extends along the entire parison length [1]. Although less flexible than dynamic methods, they can be combined with them to create highly complex thickness distributions.

After extrusion, the molds close around the parison. Internal pressure causes the plastic to expand until it fully conforms to the cavity, where it cools and solidifies. Once the material has set, the molded part is ejected and can be post-processed.

### 1.2. Simulating the Structural Performance of Blow-Molded Parts

To accurately simulate the structural performance of blow-molded products, the wall thickness distribution must first be determined through a blow-molding simulation. This distribution is governed by the process parameters that define the applied wall thickness control mechanisms. The resulting thickness field is then used as input for a subsequent structural analysis, enabling a realistic prediction of the product’s mechanical performance. Various works have been conducted in this field [3,4,5,6].

Simulating the extrusion blow molding process involves two key tasks. First, the extrusion of the parison must be modeled, taking into account mechanisms such as plastic swelling and elongation, both of which significantly influence the resulting wall thickness distribution. Tanifuji [7] provides a detailed description of this process step, highlighting the main factors that govern the final wall thickness and, consequently, its effect on the structural performance of the finished product.

Once the wall thickness distribution has been determined through blow molding simulation, the results serve as the input for virtual product testing. These tests evaluate quality-relevant parameters. For packaging applications, two testing methods are particularly common: the top-load test and the internal pressure test.

In the top-load test [8], the product is subjected to a compressive load while the resulting force–displacement relationship is recorded. This produces the top-load curve; its maximum before the first significant decrease in load indicates the load at which the structure fails, referred to as the top-loading force. This value is a key indicator of the product’s load-bearing capacity. Commonly, the reason for a sudden loss in the stability of thin-walled structures is that the product fails by buckling. As this is a stability problem and involves a complex interplay between nonlinear material and geometry as well as contact, it generally remains a challenge to simulate this behavior. Nguyen [9] demonstrates how such tests can be modeled numerically. In the context of this paper, the significant decrease is defined as the point on the curve where the first drop of at least 3% occurs.

In addition to the top-load test, the internal pressure test is commonly used to evaluate structural stability under internal pressurization. In this test, the deformation of the product’s geometry is monitored as internal pressure increases [10,11].

Since product testing is typically performed after blow molding simulation, both steps are often combined into an integrated workflow [10,11,12,13]. In such workflows, the parison is first generated and inflated, after which the resulting wall thickness distribution is automatically transferred onto a finite element mesh for structural analysis. Integrating this workflow into an optimization routine is a logical next step, enabling systematic improvements to product performance.

### 1.3. Optimization of Blow-Molded Products

The optimization of blow-molded parts presents a significant challenge due to the high dimensionality of the design space. Since the thickness of each finite element is treated as a design variable, optimizing the wall thickness distribution can involve several thousand parameters. These complexities underscore the importance of selecting an optimization algorithm capable of efficiently handling large-scale, nonlinear parameter spaces.

Several optimization approaches for blow-molded products have been reported in the literature, each addressing different design objectives. Gauvin [10] minimized product weight while satisfying performance constraints using a sequential, gradient-based algorithm. Huang used an genetic algorithm in order to homogenize the products thickness [14]. Attar [15] also aimed to reduce weight, with the additional goal of achieving a uniform wall thickness distribution, applying a gradient-based optimization scheme. Wang [16] pursued similar objectives by modifying the parison in discrete segments to achieve both weight reduction and thickness uniformity, employing an iterative update scheme that resembles a condition-based optimization procedure.

One algorithm particularly well suited for large-scale, nonlinear, and constrained optimization problems is the method of moving asymptotes (MMA). MMA is an iterative, gradient-based algorithm that constructs a convex and separable approximation of the objective function and constraints in each iteration [17,18]. Its ability to handle high-dimensional problems makes it a suitable candidate for optimizing wall thickness in blow-molded products.

**Figure 1:**
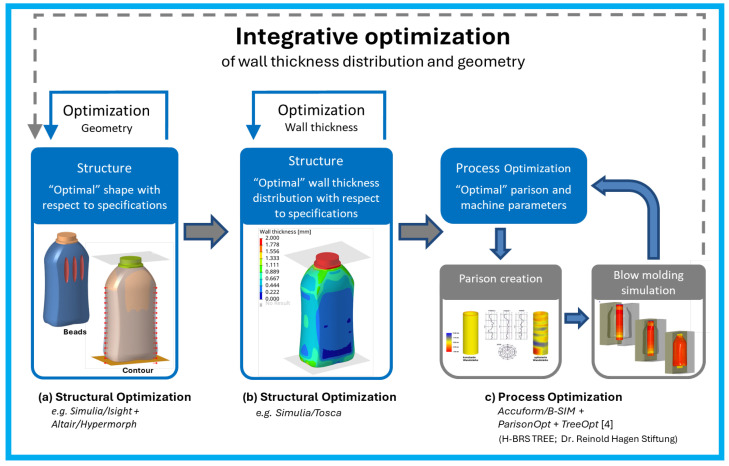
Illustration of the integrative optimization workflow [19].

This work is part of a larger research project [19] that follows a multi-step optimization workflow. First, the component’s geometry is structurally optimized. Second, the wall thickness distribution is optimized based on this geometry. At this stage, the manufacturing limitations associated with manipulating the parison’s wall thickness are not yet considered. The result of this step represents the ideal wall thickness distribution. Finally, the blow molding process is optimized by tuning machine parameters to approximate the parison’s wall thickness distribution while ensuring manufacturability. The overall process is illustrated in Figure 1.

The focus of this paper is on improving the second step of the workflow, where methods are developed and tested to optimize the wall thickness distribution. The emphasis is therefore on determining the product’s ideal wall thickness distribution. In a subsequent analysis, the result of this step will be used to optimize the machine parameters to generate a parison that, in its inflated state, resembles the product’s ideal wall thickness distribution.

### 1.4. Sensitivity Analysis in Structural Optimization

When applying gradient-based optimization, it is essential to compute the derivatives of performance measures with respect to structural parameters. These derivatives are referred to as sensitivities. Methods for computing design sensitivities can be broadly classified into finite-difference-based approaches, as well as direct and adjoint methods [20]. While finite-difference-based approaches are the most broadly applicable, especially for black-box models, they often incur higher computational costs and may suffer from numerical noise. Direct sensitivity analysis is most efficient for problems with many design responses but few design variables, whereas adjoint sensitivity analysis is preferred when there are many design variables and only a few responses.

Finite difference methods estimate sensitivities by perturbing each design variable and calculating the resulting changes in the performance measure. Although straightforward to implement, these methods become computationally expensive for high-dimensional problems. The direct method computes sensitivities by analytically differentiating the system equations, making it suitable for problems with few design variables and many responses. In contrast, the adjoint method solves an auxiliary system, requiring only two evaluations of the governing equations—independent of the number of design variables [21]. This makes it particularly efficient for large-scale optimization tasks.

In order to optimize the wall thickness distribution, it is treated as the design variable, and the top-load force serves as the performance measure to be maximized. Since the optimization problem involves a large number of design variables and a single objective function, the adjoint sensitivity method is the most suitable approach.

## 2. Research Problem and Objectives

**Figure 2:**
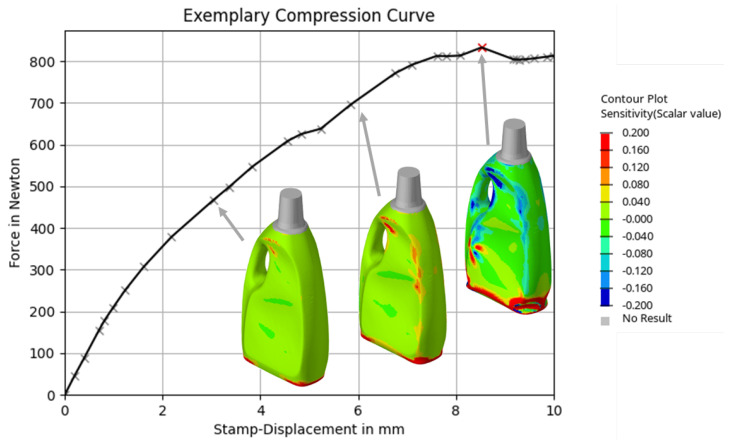
Illustration of the sensitvity of the wall thickness distribution at three deformation states with regard to the compression force.

Figure 2 illustrates the top-load curve of the product. In addition, the corresponding deformation states at different stamp displacements are shown. The color scale indicates the sensitivity of the top-load value with respect to the wall thickness distribution. Notably, the sensitivity does not scale linearly with the compression. At some points it even changes from a positive to a negative correlation (and vice versa), highlighting the nonlinear nature of the optimization problem.

As a result, the sensitivities at different deformation states yield different MMA approximations, leading to varying optimization results. This observation raises the question of how sensitivity information should be transferred to the optimization algorithm in order to efficiently optimize blow-molded products and maximize their top-load force.

Two general schemes can be envisioned for extracting sensitivities to be used in the optimization algorithm. The first involves calculating a single sensitivity value at a predefined point on the top-load curve. For example, at the maximum top-load value or at a specific stamp displacement. The second approach analyzes the transient sensitivity values mathematically to construct a substitute sensitivity vector. This vector combines information from sensitive regions across different deformation states into a single representation, providing a more holistic view of the system’s sensitivity profile.

In the following sections, different sensitivity extraction schemes are developed, together with an optimization framework into which these schemes are integrated and subsequently analyzed for their effectiveness.

## 3. Methodology

### 3.1. Sensitivities Extraction Schemes

**Figure 3:**
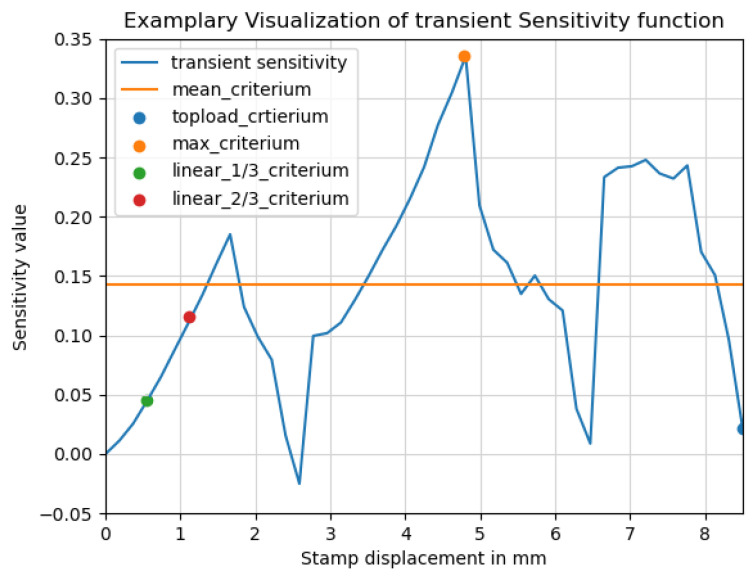
Visualization of the sensitivity value of one element during the compression experiment.

To illustrate the nonlinear behavior of an element’s sensitivity during the compression experiment, this relationship is depicted in the Figure 3. The x-axis represents the stamp displacement, while the y-axis shows the corresponding sensitivity values. This transient sensitivity function has to be reduced to a scalar value so this information can be used in an optimization procedure. In order to reduce it, various extraction schemes have been developed. Their names and the values that the methods achieve are marked in the figure. In the following paragraphs, these schemes are described in more detail.

The initial slope of the compression curves is quite linear. With a continuing compression path, more and more non-linearities emerge and the path of the curve deviates from the linear path. Because of the not-so-dominant nonlinear behavior, the compression inside of this linear part can be calculated with less computational effort. We therefore extracted the sensitivity within the initial linear slope of the top-load curve. The length of the linear region, and consequently the stroke travel for each experiment, is defined by analyzing the initial compression curve. This information is used as a 13**-criterion** and 23**-criterion**, where the sensitivities are extracted at the corresponding fraction of that value. This approach reduces computational effort due to the shorter stroke travel and the more predictable behavior of the linear region.

Next, the sensitivity is extracted at the top-load point, determined using the previously mentioned **top-load criterion**. These simulations are more complex due to the nonlinear behavior near the point of maximum load, particularly in areas where buckling occurs. In addition to generally stiffening the product, this approach is expected to stiffen the area in which buckling causes a reduction in the top-loading force.

One risk associated with the first two sensitivity extraction schemes is that they each use a single sensitivity value at a specific deformation state. In a highly non-linear context, such as in the top-load testing of blow-molded products, it is uncertain whether the exact deformation state at which the sensitivity was originally extracted will be reached again, potentially causing a decrease in compressive load at an earlier point in the curve. Therefore, for the following two criteria, sensitivities are combined from multiple points along the compression curve.

In the next criterion, the mean sensitivity of all deformation states, up to the top-load value, is calculated by calculating the average of all points in the transient sensitivity function. By using this **mean criterion**, a “substitute sensitivity” that represents a compromise that is most similar toward all available sensitivities is calculated and used for the optimization.

For the final criterion, the maximum value of each transient sensitivity function is used to create the reduced sensitivity. This **maximum criterion** combines the most sensitive states for each element throughout the entire loading process, leading to the top-load value and resulting in the stiffening of areas that are most prone to buckling at any point in the loading sequence.

In summary, four sensitivity extraction methods have been developed. The first method extracts sensitivities from specific points within the initial linear region of the compression curve, reducing computational effort. The second method focuses on extracting sensitivity at the top-load point, capturing the product’s behavior under maximum load conditions. The third method uses the mean sensitivity of all increments up to the top-load value, representing the average influence of all calculated sensitivities. Lastly, the fourth method selects the maximum sensitivity for each element, targeting the most critical deformation points to improve product robustness. Optimizations that implement these criteria are conducted in the following sections and will then be evaluated to determine to what extent the previous assumptions hold and which method works most efficiently.

### 3.2. Development of the Optimization Workflow

**Figure 4:**
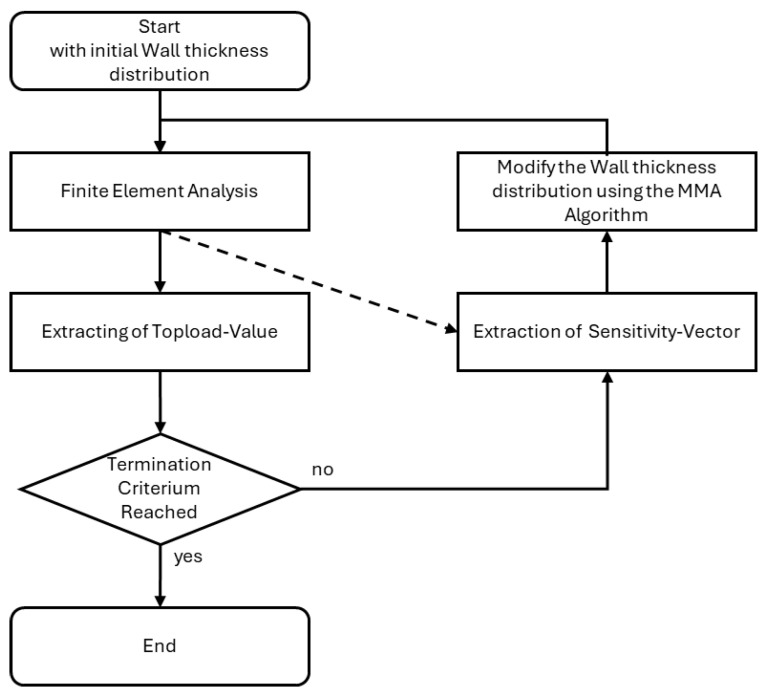
Visual representation of the optimization process. After the optimization process is started, the FEM analysis is executed. The main loop of the optimization consists of the extraction of the top-load value and of the sensitivities from the results file, after which the product’s wall thickness is adapted and the experiment is repeated.

In order to optimize the wall thickness distribution of blow-molded packaging products, the following optimization process was designed (see Figure 4). It starts with a predefined wall thickness distribution, which can be uniform or nonuniform. As a first step, the Finite Element Problem is solved. Routines were implemented that continuously check whether the top-load has been reached in the running simulation. If that is the case, the simulation is terminated as all the information necessary for the optimization has been calculated. For this purpose, the top-load condition was defined as the state just before the load drops by 3%. The achieved top-load value is then extracted from the FEM results database and represents the objective functions outcome which is to be maximized.

In each iteration, the sensitivity of the top-load value with regard to the wall thickness is extracted and stored in a column matrix. Since this sensitivity is calculated at each increment, the developed sensitivity extraction schemes (see Section 3.1) are implemented here. To optimize NLopt, a free/open-source library for nonlinear optimization has been used [22].

Because blow-molded packaging products are generally symmetric, a geometric restriction has been implemented to enforce symmetry in the wall thickness distribution. To achieve this, the sensitivities calculated by the FEM solver are filtered to create a symmetric sensitivity field. Before passing the sensitivity vector to the MMA algorithm, the values of each pair of symmetric elements are compared, and the more sensitive value is chosen for both elements. Using this procedure and starting with an already symmetric wall thickness distribution ensures that the wall thickness distribution remains symmetric throughout all iterations.

Furthermore, in this step, the mass of the product is calculated, as is the sensitivity of the product’s mass toward the wall thickness distribution. These values are then used to modify the wall thickness of each element to improve product performance using the MMA algorithm under the constraint that a predefined mass cannot be surpassed and prescribed minimum and maximum thickness limits. After adjusting the wall thickness, the FEM evaluation is repeated, completing the optimization loop.

In summary, an optimization procedure has been developed that solves the FEM problem. The iteration’s objective function value is calculated by analyzing the top-load curve. The transient sensitivity of each element is reduced to a scalar value by one of the previously described sensitivity extraction schemes. Using the MMA algorithm, the wall thickness distribution is shifted for the next iteration so that it remains within predefined limits. Furthermore, the product’s mass is constrained so that it does not exceed a predefined maximum and symmetry is enforced.

### 3.3. Numerical Experiments

The findings of this study are intended to be universally applicable to extrusion blow-molded products. To support this, optimizations are performed on three different geometries that represent common blow-molded packaging designs. This approach ensures that conclusions are not limited to a single design, but instead reflect a broader range of products, making them more widely applicable across the blow molding sector.

**Figure 5:**
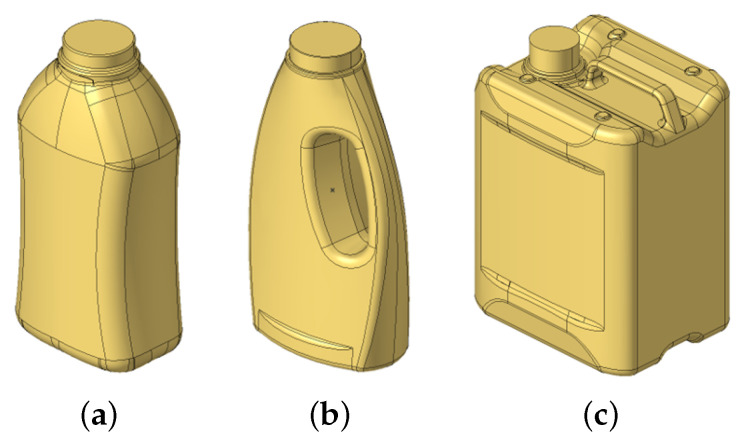
Illustration of the geometries used (**a**) Illustration of the waisted bottle. (**b**) Illustration of the handled bottle. (**c**) Illustration of the canister. (Products are not depicted to the same scale).

The first model represents a waisted bottle (see Figure 5a), commonly used for personal care products such as shampoos or conditioners. The second model is a handled bottle (see Figure 5b), frequently used for both personal care items and household cleaning products. The third model is a canister (see Figure 5c), typically used for storing and transporting fuels, oils, and other chemicals. The filling stabilizes the products and must therefore be taken into account.

In addition to geometry, the internal liquid capacity and specified product mass differ among the models. The liquid volume was selected so that each container would be mostly filled, leaving only a small amount of air. A specific product mass was chosen based on consultation with experienced blow-molding machine operators and product designers. Based on the surface area of each product, the desired mass, and the density of high-density polyethylene (HDPE), a constant wall thickness distribution was calculated. This uniform distribution served as the starting point for all subsequent optimizations.

**Figure 6:**
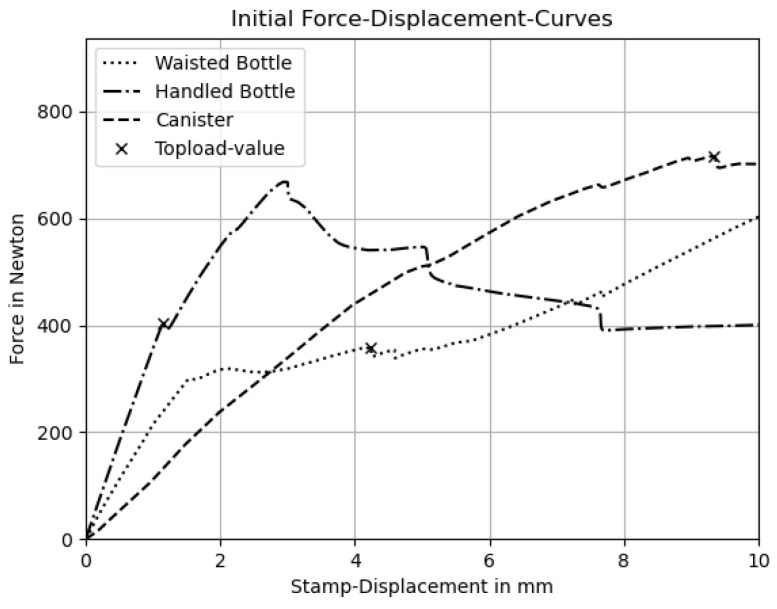
Compression curves with for the analyzed products prior to the optimization.

The finite element simulations were conducted using Dassault Systèmes Abaqus (version 2023), and the resulting compression curves were compared. For each geometry, a mesh convergence study was performed to determine an appropriate mesh density. Initial compression curves and corresponding top-load values were computed using the uniform wall thicknesses. Figure 6 shows these compression curves along with the top-load value determined using the 3% deformation criterion.

To test the criterion 3% deformation criterion, the deformation states at the top-load and immediately afterwards were visualized. Buckling was clearly visible in all three products. A further inspection of the deformation states before the identified point revealed that apart from local stiffening effects, no global buckling events were present, proving that the criterion can be effectively used to determine the top-load force.

### 3.4. Results of Linear 13 and 23-Criterion

The initial slope of the compression curve became noticeably steeper after applying the linear optimization scheme, indicating increased stiffness during the early stages of loading. However, this increase in stiffness did not generally lead to improved top-load values. A notable exception was observed in the handled bottle, where the top-load force increased by approximately 50%. Further analysis revealed that the load drop responsible for triggering the top-load criterion in the unmodified bottle was significantly reduced in the optimized version. This behavior is considered an outlier, and the assumption that increased stiffness would consistently result in higher top-load values did not hold in general.

**Figure 7:**
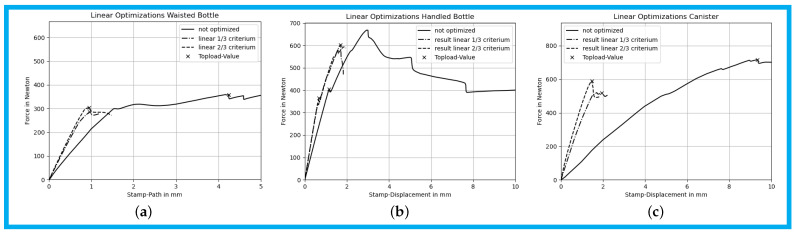
(**a**) Comparison of the initial and optimized compression curves for the waisted bottle. (**b**) Comparison of the initial and optimized compression curves for the handled bottle. (**c**) Comparison of the initial and optimized compression curves for the canister.

A comparison of the unmodified and optimized products, shown in Figure 7a–c, reveals that the compression states differed significantly. Buckling occurred in different regions, indicating that the applied linear criteria influenced not only the stiffness but also the overall deformation behavior.

**Figure 8:**
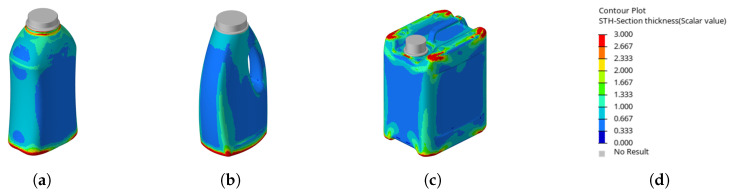
Illustration showing the resulting wall thickness distributions (im mm) using the optimizations using the **linear** 13 criterion on the distinct geometries (**a**). Resulting wall thickness on the waisted bottle (**b**). Resulting wall thickness on the handled bottle (**c**). Resulting wall thickness on the canister (**d**). Scale for reference.

When examining the optimized wall thickness distributions, it becomes apparent that the linear sensitivity extraction scheme tends to shift material toward the edges and corners of the product. This outcome is intuitive, as the applied criterion does not involve buckling; consequently, the structure cannot be selectively reinforced at potential buckling locations. Figure 8 illustrates the wall thickness distribution resulting from the optimization scheme based on the **linear** 13**-criterion**.

It is assumed that the reason for the suboptimal results is the relatively large spatial separation between the sensitivities, which were extracted at the very beginning of the simulation, and the top-load value. The increasingly strong nonlinearities that emerged as the simulation progresses toward the top-load value render these sensitivities unrepresentative of the overall problem, restricting their validity to the initial, nearly linear part of the top-load curve. The fact that the initial linear part of the top-load curve was stiffened with all products proves that point. Such behavior may be advantageous in optimization problems with different objectives, such as the optimization of hazardous materials containers, which are generally designed to be as stiff as possible.

### 3.5. Results of Top-Load Criterion

**Figure 9:**
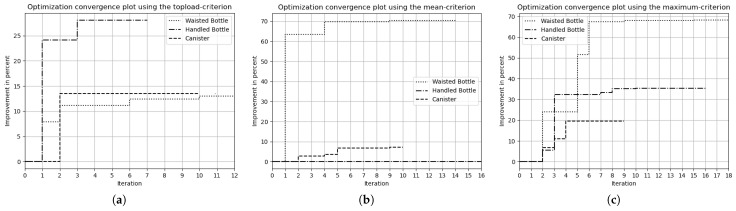
Graphs showing the convergence plots of the conducted experiments using the (**a**) top-load criterion, (**b**) mean-criterion, (**c**) maximum criterion.

The optimizations based on the **top-load criterion** resulted in improved product performance. To visualize the progress of the optimization, the archived top-load value of each iteration is plotted in Figure 9a. For all geometries, the optimization process converged after only a few iterations.

**Figure 10:**
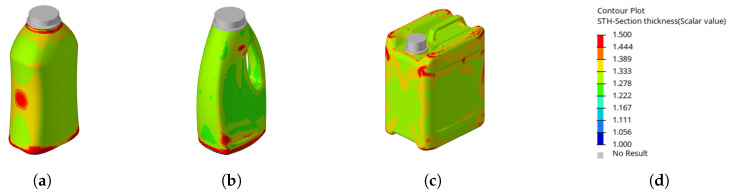
Illustration showing the resulting wall thickness distributions (in mm) using the optimizations using the **top-load criterion** (**a**). Resulting wall thickness on the waisted bottle (**b**). Resulting wall thickness on the handled bottle (**c**). Resulting wall thickness on the canister (**d**). Scale for reference.

When examining the optimized wall thickness distributions (see Figure 10), it became apparent that the material shifted differently compared to its behavior under the linear 23 **criterion**. In this case, regions where buckling triggers the top-load criterion are locally stiffened, which delays the onset of buckling and consequently increases the top-load value.

### 3.6. Results of Mean Criterion

Using the **mean criterion**, the optimization of the waisted bottle resulted in a significantly improved top-load value. For the other two products, however, this criterion did not lead to performance improvements (see the convergence plot in Figure 9b). An analysis of the deformation states revealed that the waisted bottle gradually deformed along the compression path, resulting in a sensitivity field that remained consistent over a larger number of increments. In contrast, buckling in the canister and handled bottle occurred more spontaneously, generating localized regions of high sensitivity for only a short duration. It is assumed that, in these cases, the sensitivities were smoothed out over time, reducing their effectiveness in guiding the optimization.

### 3.7. Results of Maximum Criterion

In contrast, the **maximum criterion** yielded improved wall thickness distributions for all three products. However, convergence toward the optimum required more iterations compared to the optimization using the top-load criterion (see Figure 9c).

**Figure 11:**
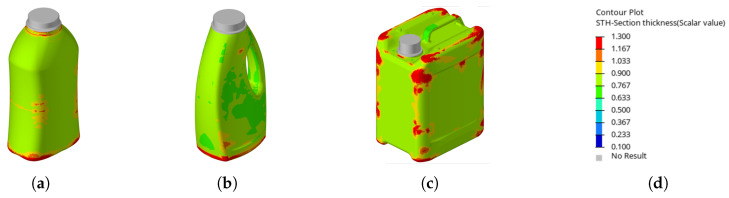
Illustration showing the resulting wall thickness distributions (in mm) with optimizations using the **maximum criterion** (**a**). Resulting wall thickness on the waisted bottle (**b**). Resulting wall thickness on the handled bottle (**c**). Resulting Wall thickness on the canister (**d**). Scale for reference.

Inspection of the wall thickness distribution revealed that the material shifted in a similar manner to the top-load criterion, but with more localized thickened regions (see Figure 11). This behavior is likely due to the fact that the maximum criterion accounts for all local buckling events—even those that do not trigger the 3% threshold—resulting in a stiffer product with a higher top-load capacity.

### 3.8. Analysis of Optimization Results

**Figure 12:**
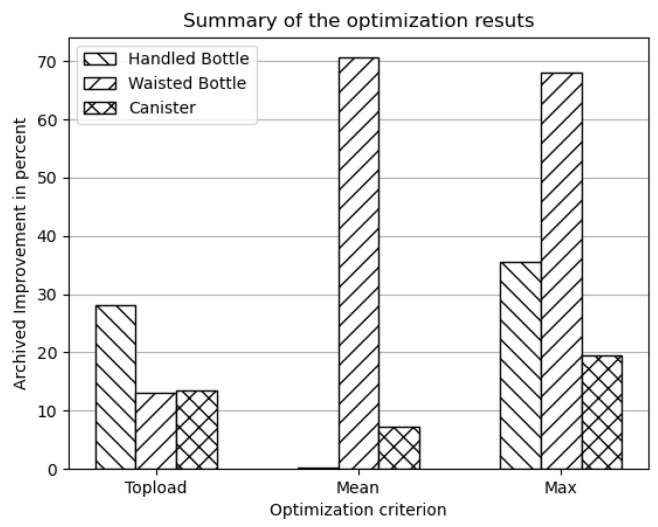
Summary of the optimization results. Since the results of the linear optimization scheme did not increase the top-load force, these results are omitted from this summary.

In summary, the different optimization approaches demonstrated varying levels of effectiveness in improving the load-bearing capacity of blow-molded packaging products, while achieving a range of distinct optimal wall thickness distributions. The perceived improvement associated with each tested criterion is illustrated in Figure 12.

No single approach consistently delivered the best results. The top-load criterion consistently led to improved products, but the improvement was less pronounced compared to those achieved under other criteria. This is assumed to be due to the fact that all sensitivities are extracted at the top-load value. Similarly to the linear criteria, where the transient progression of the sensitivity function was not taken into account, the top-load criterion also considers only the sensitivity at the end of the sensitivity function, neglecting its overall progression. However, since the extracted sensitivity values describe the system state immediately before the failure criterion is triggered, from iteration to iteration, this point moves toward later compression states and improved products are nevertheless obtained.

The two remaining criteria derive a sensitivity value from the entire sensitivity function. The mean criterion smoothens out sudden changes in sensitivity, resulting in improved performance only when buckling occurs over an extended period. Since buckling typically arises spontaneously, this behavior is not considered advantageous for the respective application. In contrast, the maximum criterion captures the highest sensitivity peak for each element, irrespective of its individual occurrence. This approach consistently produces very good results.

The linear criterion required the least computational effort, as only the initial linear part of the compression curve was simulated. The top-load and mean criteria converged faster than the maximum criterion but are not considered suitable for practical use. Optimizations with the maximum criterion required the most effort, yet convergence was reached in fewer than ten simulations per geometry, corresponding to a manageable runtime of a few tens of hours on a modern workstation. Since the procedure is intended to be applied only once during the product development cycle, a detailed runtime analysis was not performed.

## 4. Conclusions

This paper investigates approaches for condensing transient sensitivity values from nonlinear finite element analyses into a single scalar measure for use in optimization schemes. Several reducing methods were developed and evaluated within a dedicated, self-developed optimization framework to assess their influence on the optimization outcome.

While not all methods proved beneficial within the optimization framework, two approaches emerged as particularly effective. Optimization restricted to the initial linear region of the top-load curve produced significantly stiffer structures while requiring less computational effort than alternative methods. In addition, both the top-load criterion and the maximum criterion yielded notable improvements in wall thickness distribution with respect to top-load performance. However, as the maximum criterion consistently provided superior results, it is recommended for future investigations.

Based on the findings, the following design guidelines are proposed: When the objective is to achieve a high degree of stiffness and minimize deformation under load, optimization using the linear criterion is recommended. This approach is particularly suitable for applications such as canisters for hazardous materials, which are evaluated not only for top-load capacity but also for long-term creep resistance, both of which benefit from increased stiffness. In contrast, when the goal is to maximize the top-load capacity, the maximum criterion should be applied. This strategy is especially appropriate for consumer packaging, where the primary focus lies in enhancing load-bearing performance while minimizing material usage.

## 5. Outlook

This study forms part of a broader optimization framework under development, aiming to enhance structural performance while reducing material consumption. While the present work focused on increasing the product’s top-load capacity through optimization of the material distribution, future developments will extend the workflow to industrial practice by simultaneously optimizing geometry and material distribution, emphasizing material efficiency alongside performance. Instead of maximizing top-load capacity, the objective will be to minimize material demand while ensuring compliance with a minimum permissible top-load value. In addition, process-related constraints from the blow molding inflation stage will be incorporated, as the attainable wall thickness distribution is strongly governed by parison and process parameters. Explicitly accounting for these restrictions will ensure that optimized designs remain structurally sound, resource-efficient, and feasible in manufacturing practice.

## Data Availability

The data that support the findings of this study are available from the corresponding author upon reasonable request.

## Abbreviations

The following abbreviations are used in this manuscript:

MMA	Method of Moving Asymptotes
FEM	Finite Element Method
HDPE	High-Density Polyethylene
